# NLRP3-mediated pyroptosis in cardiovascular disease: from molecular mechanisms to therapeutic targets

**DOI:** 10.3389/fphar.2026.1901368

**Published:** 2026-09-10

**Authors:** Qiuyu Xu, Wei Sun, Linwei Li, Ning Liu, Bin Liu

**Affiliations:** Department of Cardiology, The Second Hospital of Jilin University, Changchun, China

**Keywords:** cardiovascular diseases, inflammation, molecular mechanisms, NLRP3 inflammasome, pyroptosis, therapeutic targets

## Abstract

Globally, cardiovascular disease has consistently been the leading cause of death. Its pathogenesis is complex and involves interactions among multiple factors, with inflammatory mechanisms garnering increasing attention. Pyroptosis—pro-inflammatory programmed cell death—is categorized into classical, non-classical, and other pathways. Among these, the inflammasome-induced classical pathway has been rigorously studied. Recent findings show that pyroptosis is essential for the development and course of cardiovascular conditions, especially atherosclerosis, myocardial infarction, and heart failure. This review analyzes current evidence on the molecular underpinnings and clinical significance of NLRP3-induced pyroptosis in cardiovascular disease. This review addresses a critical gap in current anti-inflammatory therapies for cardiovascular disease by bridging molecular insights with translational innovations. It advocates for mechanism-driven clinical trials and the use of pyroptosis-modulating techniques to develop next-generation therapies, aiming to provide a theoretical framework for subsequent studies and treatment approaches.

## 1 Introduction

Pyroptosis was first defined in the early 1990s, when Zychlinsky et al. (1992) identified this form of cellular death in *Shigella flexneri*-infected mouse macrophages. Initially, this form of cell death was considered apoptosis, but it was subsequently found to be a distinct type. In 2001, Salmonella-induced cell death was realized to be entirely distinct from apoptosis; Cell membrane integrity was lost as a result of early *Salmonella* infection, and caspase-1 activation was necessary for the resulting cell death (Boise and Collins, 2001). In the same year, to characterize this kind of cell death, Cookson et al. coined the term “pyroptosis.” The word, which refers to pro-inflammatory programmed cell death, comes from the Greek words “pyro” (fire/heat) and “optosis” (to-sis, falling) (Cookson and Brennan, 2001). This was the first formal definition of pyroptosis, intended to distinguish it from apoptosis.

One important component of the conventional pyroptosis pathway is the NLRP3 inflammasome. The receptor (NLRP3), adaptor (apoptosis-related small protein [ASC]), and effector (half-caspase-1) proteins make up the NLRP3 inflammasome, a multicomponent cytoplasmic protein complex. Pattern recognition receptors (PRRs) on the cell surface, such as Toll-like receptors (TLRs) and interleukin-1 receptor (IL-1R), recognize extracellular pathogen-associated molecular patterns (PAMPs) and danger-associated molecular patterns (DAMPs), thereby activating the nuclear factor kappa-B (NF-κB) signaling pathway. Activation of this pathway subsequently promotes the transcription and translation of NLRP3 inflammasome-related genes, including NLRP3, ASC, and caspase-1.

**FIGURE 1:**
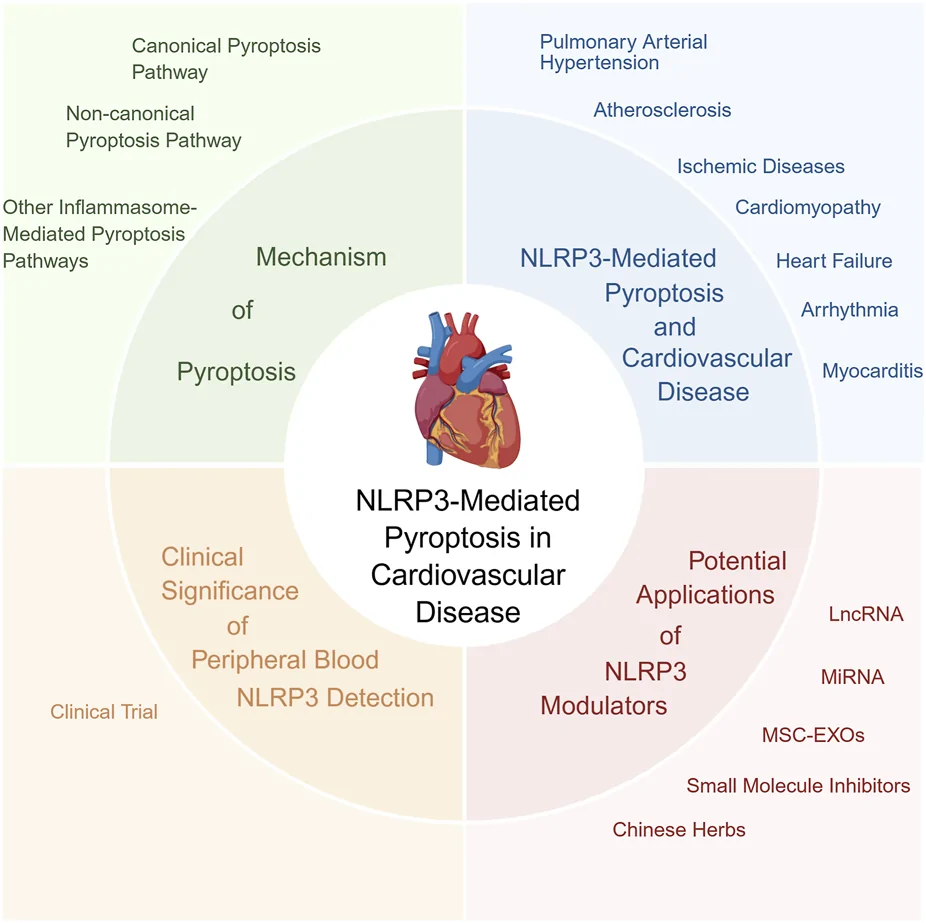
Xu, Q. (2026). NLRP3-Mediated Pyroptosis in Cardiovascular Diseases. Created in BioRender. https://BioRender.com/et5s1au.

There is mounting evidence linking pyroptosis to the development, course, and management of cardiovascular diseases. This review aimed to summarize research advances on NLRP3-mediated pyroptosis in cardiovascular diseases and potential treatment applications (Figure 1). Additionally, the potential of the NLRP3 inflammasome as a diagnostic biomarker for cardiovascular diseases is discussed.

## 2 Mechanism of pyroptosis

### 2.1 Classical pathway

Signaling cascades leading to pyroptosis are initiated by the inflammasome, which cleaves gasdermin D (GSDMD) and releases Inflammatory cytokines, including IL-1β and IL-18 (Yu et al., 2021). Sensor proteins, adaptor proteins, the proenzyme caspase-1, and ASC make up the multimolecular complex known as the inflammasome. Sensor proteins include AIM2-like receptors (ALRs) or sensor proteins of the NLR family with nucleotide-binding domains and leucine-rich repeats (Vanaja et al., 2015). Currently, the inflammasome sensors NLRP1, NLRP3, NLRC4, pyrin, and AIM2 can construct classic inflammasomes (Martinon et al., 2002), with the NLRP3 inflammasome as the most extensively studied. In cardiovascular disease, the activation of the NLRP3 inflammasome exhibits a marked preference for endogenous “sterile” danger signals, which distinguishes it pathologically from NLRP1, NLRC4, and AIM2. Key pathogenic factors in atherosclerosis, myocardial infarction, and heart failure—such as cholesterol crystals, oxidized low-density lipoprotein, abnormal blood flow shear stress, mitochondrial reactive oxygen species, and extracellular ATP—are all classical NLRP3 agonists; they activate the NLRP3 inflammasome through common upstream signaling pathways, such as triggering potassium efflux, lysosomal rupture, or mitochondrial damage (Zhaolin et al., 2019; Li et al., 2018). AIM2 recognizes double-stranded DNA released into the cytoplasm by dying cells, thereby promoting the release of IL-1β and IL-18 (Onódi et al., 2021). NLRC4 specifically recognizes bacterial flagellin and plays a major role in host defense against infection; there is very little evidence of its role in non-infectious CVD processes (Miao et al., 2006). It is evident that the activation criteria for these two pathways are relatively simple, and their overall contribution to pathogenesis is far lower than that of the NLRP3 inflammasome. Therefore, there is indeed a genuine “NLRP3-dominant pathological feature” in CVD, wherein various danger signals derived from metabolic disorders and tissue damage converge on a single NLRP3-dependent inflammatory pathway, forming the core loop driving disease progression.

The NLRP3 inflammasome is composed of three core components: the sensor NLRP3, the adaptor protein ASC (also known as PYCARD), and the effector protein caspase-1. The sensor NLRP3 is a tripartite protein, comprising, from the N-terminus to the C-terminus, a pyrin domain (PYD), a central NACHT domain (found in NAIP, CIITA, HETE, and TP1), and a leucine-rich repeat (LRR) domain. The adaptor protein ASC contains two domains: an N-terminal PYD and a C-terminal caspase recruitment domain (CARD). The effector protein caspase-1 contains an N-terminal CARD, a central large catalytic subunit (p20), and a C-terminal small catalytic subunit (p10) (Boucher et al., 2018).

The NLRP3 inflammasome activation process comprises initiation and activation phases. During initiation, the NLRP3 inflammasome, caspase-1, and pro-IL-1β are expressed at higher levels, and the NLRP3 inflammasome undergoes post-translational changes. This step can be triggered by pattern recognition receptor-mediated signaling, which can be initiated by the recognition of various pathogen-associated molecular patterns (PAMPs) or damage-associated molecular patterns (DAMPs) (such as toll-like receptor 4 [TLR4]), transcriptional control, and post-translational modification processes (Franchi et al., 2009; Tannahill et al., 2013). Concurrently, the NLRP3 inflammasome activation is induced through pathways such as potassium (K+) or chloride (Cl−) efflux, calcium (Ca^2+^) flux, mitochondrial damage (reactive oxygen species [ROS] burst, mitochondrial DNA release), metabolic changes, and trans-Golgi disassembly (Xu and Núñez, 2023).

After NLRP3 inflammasome assembly is completed, it oligomerizes through homotypic interactions between its NACHT domains. The oligomerized NLRP3 inflammasome then recruits ASC via homotypic PYD-PYD interactions. Subsequently, multiple ASC filaments assemble into a single macromolecular focus, known as the ASC speck (Masumoto et al., 1999). The assembled ASC acts as a link connecting the sensor to the effector; it attracts caspase-1 and induces the autolysis of nearby caspase-1 into a fully hydrolytic active state (Ross et al., 2018; Lu et al., 2014). Next, activated caspase-1 cuts pro-IL-1β and IL-18, turning them into mature cytokines, and activates GSDMD, a crucial effector protein involved in pyroptosis (Schroder and Tschopp, 2010; Vande Walle and Lamkanfi, 2024), ultimately completing the pyroptosis process.

### 2.2 Non-classical pathway

Human caspases-4/5 lack an upstream sensor complex and instead bind directly to intracellular lipopolysaccharide (LPS) and lipid A with exquisite specificity and affinity (Shi et al., 2014). The caspase’s CARD domain mediates LPS binding, which promotes caspase-4/5/11 oligomerization and self-cleavage activation. GSDMD can also be proteolyzed by activated caspase-4/5/11 to generate N-GSDMD, which subsequently oligomerizes and translocates to the cell membrane, where it ultimately forms pores in the plasma membrane (Aglietti et al., 2016).

### 2.3 Other inflammasome-mediated pyroptosis pathways

#### 2.3.1 Caspase-8-dependent pathway

*In vitro* research indicates that the substrate specificities of caspase-8 and caspase-1 overlap. For instance, caspase-8 does not require the creation of an inflammasome complex or caspase-1 to directly process and activate IL-1β downstream of death receptor and TLR signaling (Maelfait et al., 2008; Bossaller et al., 2012). Concurrently, Pyroptosis can be induced by caspase-8 cleaving and activating GSDMD. For instance, *Yersinia* enterocolitica YopJ-induced death in mouse macrophages involves caspase-8-mediated cleavage of GSDMD (Sarhan et al., 2018; Demarco et al., 2020). Furthermore, under certain pathogen stimuli (such as a virus), caspase-8 can drive NLRP3 inflammasome activation, thereby inducing pyroptosis via the classical pathway.

#### 2.3.2 Caspase-3-GSDME pathway

GSDME serves as a key protein in the transition between apoptosis and pyroptosis. Researchers have identified a caspase-3 cleavage site (267DMPD270) in human GSDME and used chemotherapy drugs to activate caspase-3, thereby cleaving GSDME. The resulting membrane pore effect acts on the plasma membrane of apoptotic cells, leading to pyroptosis-like necrosis (Wang et al., 2017).

### 2.4 Potential caspase-independent pyroptosis pathways

**FIGURE 2:**
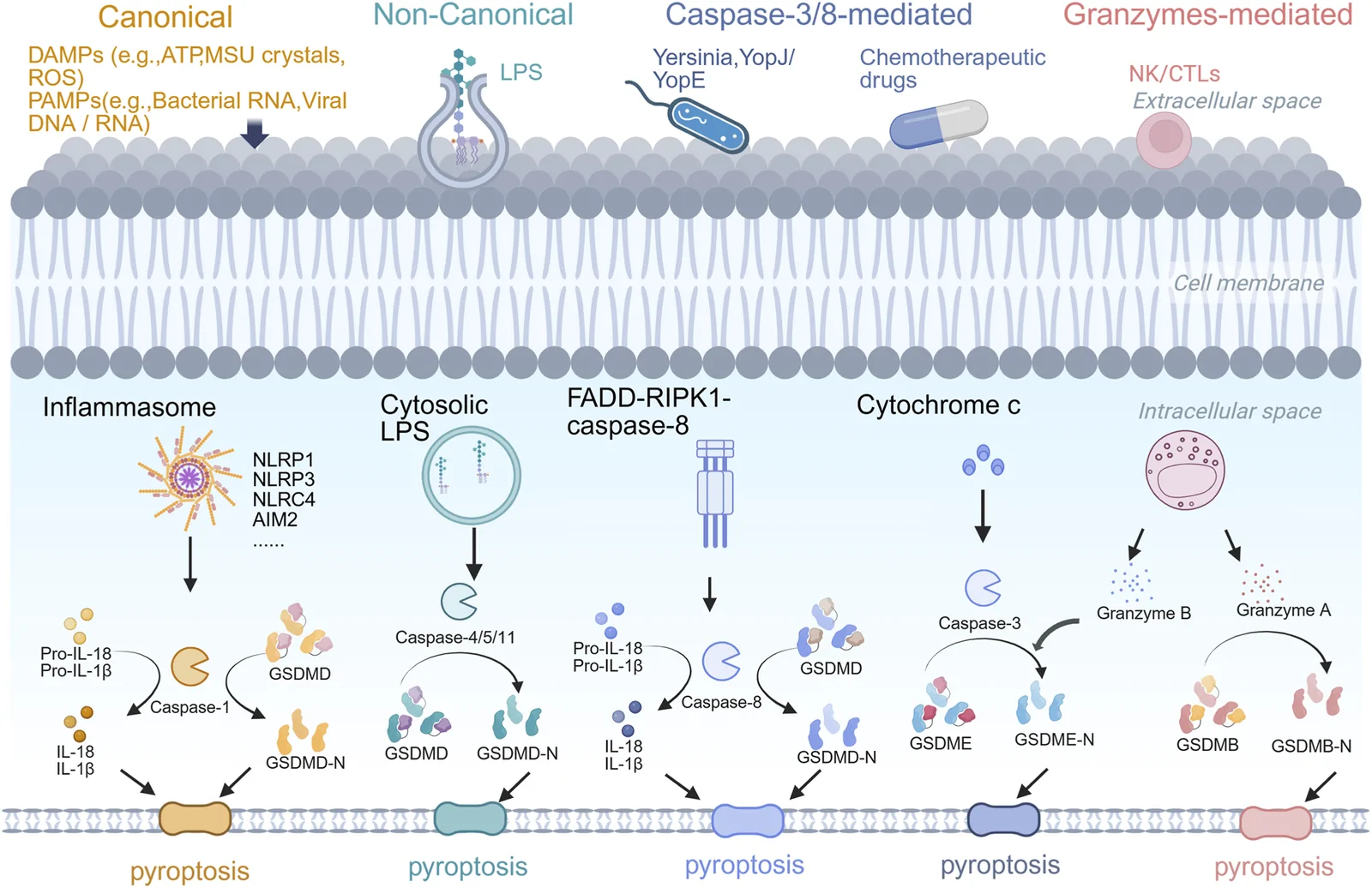
Schematic diagram illustrating the mechanism of pyroptosis. This figure illustrates the classical and non-classical signaling pathways of pyroptosis. In the classical pathway, pathogen-associated molecular patterns (PAMPs) or damage-associated molecular patterns (DAMPs) activate pattern recognition receptors (such as TLR4/NF-κB), thereby upregulating the transcriptional expression of the NLRP3 inflammasome, pro-IL-1β, and pro-IL-18. Subsequently, ASC and pro-caspase-1 are recruited; activated caspase-1 cleaves GSDMD to generate the pore-forming GSDMD-N fragment, inducing cell membrane perforation and the release of cellular contents, and also cleaves pro-IL-1β and pro-IL-18 into their mature forms, IL-1β and IL-18, thereby exacerbating the inflammatory response. In the non-classical pathway, intracellular LPS directly activates caspase-4/5 (mouse caspase-11), which in turn cleaves GSDMD and indirectly activates the NLRP3 inflammasome, amplifying inflammatory signaling. Under specific stimuli, activated caspase-3 cleaves GSDME, releasing the GSDME-NT porogenic fragment, which induces pyroptosis. The death of mouse macrophages induced by *Yersinia* YopJ and other factors can be mediated by caspase-8-induced cleavage of gasdermin D. As a new mechanism of immune killing, NK cells can deliver granzyme A into target cells via perforin. GZMA directly cleaves the GSDMB protein within target cells, releasing its N-terminal pore-forming fragment. The excessive activation of this pathway mediates cardiomyocyte pyroptosis, vascular endothelial dysfunction, and immune cell infiltration, which constitute important pathological foundations for cardiovascular diseases such as atherosclerosis, myocardial ischemia/reperfusion injury, and heart failure. Xu, Q. (2026). Schematic diagram illustrating the mechanism of pyroptosis. Created in BioRender. https://BioRender.com/7h4e1fd.

Granzyme A (GZMA), which can directly cleave GSDMB and initiate caspase-independent pyroptosis, is released by NK cells and cytotoxic T lymphocytes (Zhou et al., 2020). Additionally, GZMA can directly cleave GSDME to trigger caspase-dependent pyroptosis in target cells (Liu et al., 2020). The following figure shows the mechanisms of various pyroptosis pathways (Figure 2).

## 3 NLRP3-mediated pyroptosis and cardiovascular disease

### 3.1 Atherosclerosis

**FIGURE 3:**
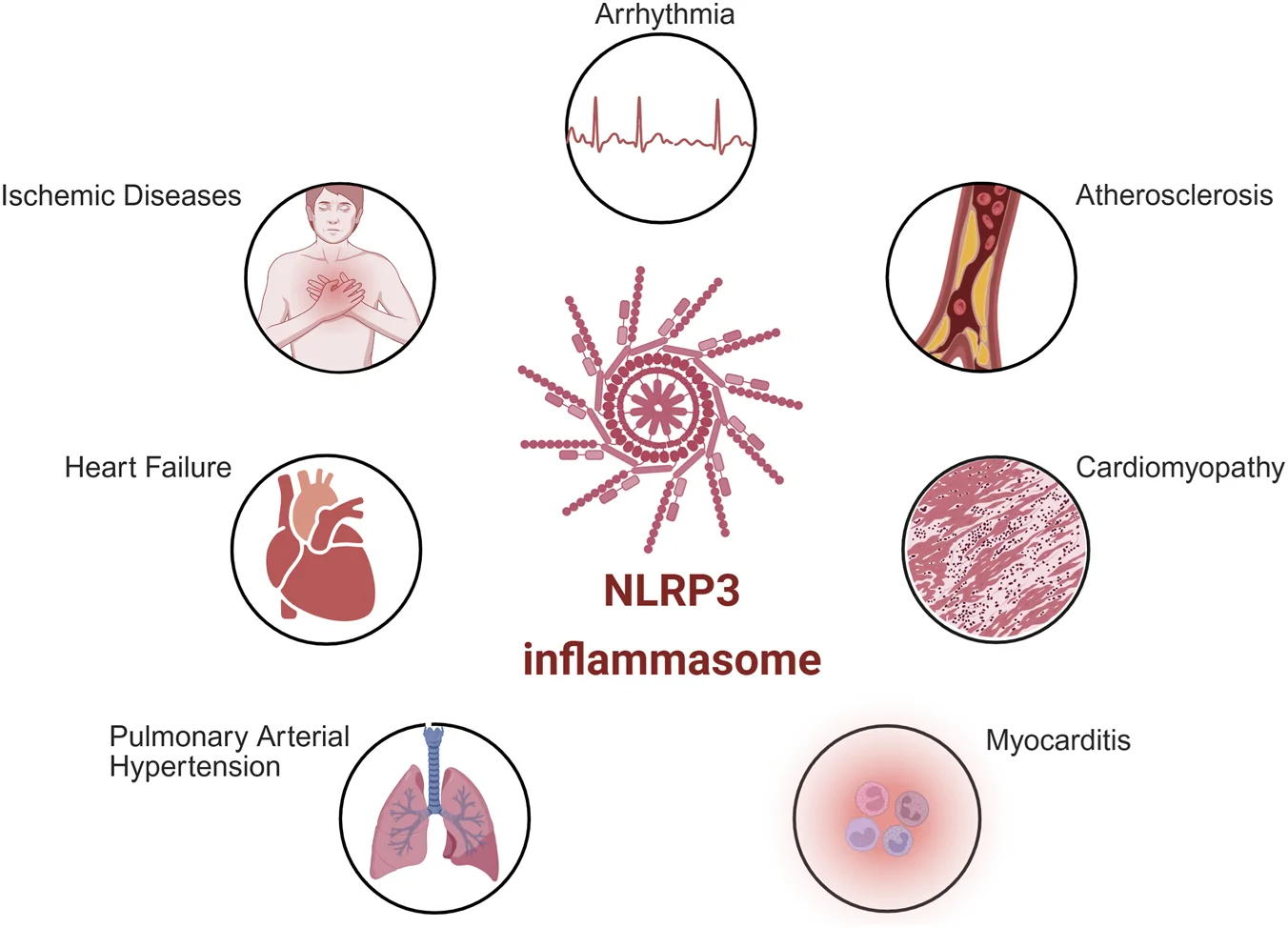
Schematic illustrating the association of NLRP3 with various cardiovascular diseases. This figure systematically summarizes the key roles of the NLRP3 inflammasome in various cardiovascular diseases and the pathological mechanisms it mediates. Overexpression of the NLRP3 inflammasome contributes extensively to the onset and progression of diseases such as atherosclerosis, myocardial ischemia/reperfusion injury, heart failure, myocardial infarction, diabetic cardiomyopathy, and atrial fibrillation by promoting the maturation of inflammatory cytokines (such as IL-1β and IL-18) and inducing pyroptosis. Xu, Q. (2026). Schematic illustrating the association of NLRP3 with various cardiovascular diseases. Created in BioRender. https://BioRender.com/vlxum93.

Atherosclerosis is considered a chronic inflammatory disease, as inflammation is centrally involved in all stages of the atherosclerotic process (Ross, 1999; Libby et al., 2011). Given the pro-inflammatory nature of pyroptosis, it is likely associated with atherosclerosis (Figure 3).

Fucoxanthin (FUCO) is reported to promote endothelial cell survival by triggering the PI3K/AKT signaling pathway, thereby inhibiting oxidized low-density lipoprotein–induced NLRP3/caspase-1-mediated pyroptosis in AECs. In addition, FUCO reduces oxidized low-density lipoprotein–induced pyroptosis in AECs by inhibiting the TLR4/NFκB/NLRP3/caspase-1 signaling pathway (Cui et al., 2023). Zhang S. et al. (2023) demonstrated that homocysteine promotes atherosclerosis by inducing NLRP3/caspase-1–mediated macrophage pyroptosis through endoplasmic reticulum (ER) stress, ER-mitochondrial coupling, and disruption of calcium homeostasis (Table 1).

**TABLE 1:** Key regulators and effector molecules of NLRP3-mediated pyroptosis in cardiovascular diseases.

Disease	Compounds or therapies	Disease model	Mechanism	Effects (promote, inhibit)	References
Atherosclerosis	FUCO	MAECs	PI3K/AKT↑→NLRP3/caspase-1↓ TLR4/NFκB/NLRP3/caspase-1↓	(−)	Cui et al. (2023)
	Homocysteine	THP-1	ER stress and ER-mitochondria coupling-induced calcium dysregulation→NLRP3/caspase-1↑	(+)	Zhang S. et al. (2023)
	BAM15	MAECs	NLRP3/ASC/caspase-1↓	(−)	Zhong et al. (2025)
	METTL3	HAECs	Enhancing the stability of the lncRNA H19 through m6A modification →NLRP3/Caspase-1↑	(+)	Tang F. et al. (2024)
	Ursolic acid	Bioinformatics analysis	CASP1,CASP8 and *IL-1β*	(−)	Huang et al. (2024)
Myocardial infarction	RBP4	mouse primary ventricular myocytes	NLRP3/caspase-1/GSDMD↑	(+)	Zhang K. Z. et al. (2021)
	ALKBH5	RCM, H9c2	NLRP3/CASP1/GSDMD↑	(+)	Cui et al. (2025)
	HIF-1α	HL-1	TUG1/NLRP3↑	(+)	Wang et al. (2022)
Myocardial ischemia-reperfusion injury	MARCH9	Mouse primary cardiomyocytes	Induces K48-linked polyubiquitination of NLRP3, promoting the proteolytic degradation of NLRP3	(−)	Lu H. et al. (2025)
	USP25	Mouse/HL-1	Inhibit the activation of the NLRP3 inflammasome by removing the K63-linked ubiquitin chain from NLRP3	(−)	Ye et al. (2025)
	Ozone	Rat	NLRP3/caspase-1↓	(−)	Xu G. et al. (2025)
	Up-frameshift protein 1	Mouse	KLF6/TXNIP/NLRP3↓	(−)	Yan et al. (2026)
Ischemic stroke	IFP35	BV2	TLR-4/NF-κB/NLRP3↑	(+)	Zhang M. et al. (2025)
	Effusol	Rat/BV2	TLR-4/NF-κB/NLRP3↓ NLRP3, caspase-1, ASC↓	(−)	Xu L. et al. (2025)
	ADSC-EVs	Rat/BV2	Gbp3/NLRP3/GSDMD↓	(−)	Wang J. et al. (2025)
	IFITM1	Mouse/BV2	NLRP3/IL-β↑	(+)	Li and Huang (2025)
	Bakuchiol	Mouse/BV2	AMPK/Nrf2↑ →TXNIP/NLRP3/Caspase-1↓	(−)	Xu Y. W. et al. (2025)
	TSFP	Rat	Inhibits the phosphorylation of p38 and ERK1/2, promotes the phosphorylation of ERK5, NLRP3/caspase-1/GSDMD↓	(−)	Wang K. et al. (2025)
	Edaravone	Rat/BV2	STING/NLRP3/GSDMD↓	(−)	Zhang Y. et al. (2025)
	Remimazolam	Rat/primary rat cortical neurons	NF-κB/NLRP3/caspase-1↓	(−)	Liu H. et al. (2025)
Ischemic kidney disease	CD8 + CD103 + iTregs	Mouse/TCMK-1	NLRP3/Caspase-1↓	(−)	Chen et al. (2024)
	G9a	Mouse/HK-2	G9a catalyzes the methylation of the lysine residue at position K262 of FoxO3a →TRIM21 mediates K48-linked ubiquitination of FoxO3a at lysine 176 →Degradation of FoxO3a↓ →NLRP3/caspase-1/IL-1β↑	(+)	Zheng et al. (2025)
	METTL3	Mouse/HK2	TIFA/NLRP3/GSDMD/IL-1β↑	(+)	Ji et al. (2025)
	PRDM16	Mouse/Boston University mouse proximal tubular cells	USP10↑→NLRP3/Caspase1/GSDMD↓	(−)	Han et al. (2025)
	Dapagliflozin	Mouse/HK-2	AMPK/SIRT1↑→NLRP3↓	(−)	Zhu et al. (2025)
	Pioglitazone	Rat/HK-2	NLRP3↓	(−)	Ye Z. et al. (2024)
	Sodium aescinate	Mouse/HK-2	AKT/NLRP3↓	(−)	Xin L. et al. (2025)
Ischemic retinal diseases	Hydrogen sulfide	Rat	NLRP3/Caspase-1/GSDMD↓	(−)	Yang et al. (2022)
	Homer1	Mouse/retinal ganglion cells	Endoplasmic reticulum stress/TXNIP/NLRP3/Caspase1↓	(−)	Lv et al. (2023)
	Stmp1	Mouse/microglia	NLRP3/caspase-1↑	(+)	Zheng et al. (2023)
DCM	HDAC6	Rat	Inhibit autophagy→NLRP3↑	(+)	Pang et al. (2023)
DM	UCP2	Mouse/H9c2	ROS/TXNIP/NLRP3/GSDMD↓	(−)	Zhang L. et al. (2025)
	USP13	Mouse	Removal of the K63-linked ubiquitin chain at K557 in NLRP3→Inhibiting the NLRP3-ASC interaction	(−)	Xu D. et al. (2025)
SICM	NEK7	Mouse/HL-1	NLRP3↑	(+)	Wen et al. (2025)
	TNIK	Mouse/HL-1	NLRP3/IL-1β↑	(+)	Yang W. et al. (2025)
	IRF1	Mouse/H9c2	NLRP3↑	(+)	Pei et al. (2026)
AF	H2	Rat/HL-1	NOX4/ROS/NLRP3↓	(−)	Zhang B. et al. (2025)
HF	FTO	H9c2	TLR4/NF-κB/NLRP3↓	(−)	Tu et al. (2024)
	DUOX1	AC16	ROS/NLRP3/caspase-1/GSDMD-N↑	(+)	Li et al. (2024)
	EAT-myocardial axis	Mouse/H9c2	NLRP3/GSDMD↑	(+)	Xia et al. (2022)
PH	Urolithin A	Mouse/hPASMC	AMPK↑→NF-κB/NLRP3↓	(−)	He et al. (2024)
	Bone morphogenetic protein receptor 2	Rat/PAEC	NLRP3/GSDME↓	(−)	Tian et al. (2025)
Myocarditis	MG53	HL-1	NF-κB/NLRP3/GSDMD-N↓	(−)	Xue et al. (2024)
	IL-37	Mouse	NF-κB/NLRP3/GSDMD↓	(−)	Sun L. et al. (2022)
	Cathepsin B	Mouse	NLRP3/caspase-1↑	(+)	Wang et al. (2018)
	Lupeol	Bone marrow-derived macrophages	αPPARα↑→LACC1/NF-κB/NLRP3/GSDMD↓	(−)	Xiong et al. (2024)

Abbreviations: ASC, apoptosis-related small protein; ADSC, adipose-derived stem cells; AF, atrial fibrillation; AMPK, adenosine monophosphate-activated protein kinase; DCM, dilated cardiomyopathy; FUCO, fucoxanthin; GSDMD, gasdermin D; HAECs, human aortic endothelial cells; HF, heart failure; HIF, hypoxia-inducible factor; IL, interleukin; MG53, Mitsugumin 53; NF-κB, nuclear factor κB; NOX, NADPH, oxidase; PH, pulmonary hypertension; RBP4, retinol-binding protein 4; ROS, reactive oxygen species; SICM, sepsis-induced cardiomyopathy; TLR, toll-like receptors; UCP., uncoupling protein; FTO, fat mass and obesity-associated protein; DUOX1, dual oxidase 1; H_2_, hydrogen; IRF1, interferon regulatory factor 1; TNIK, Traf2- and Nck-interacting kinase; NEK7, Necrotic-associated kinase 7; ALKBH5, AlkB homolog 5; RCM, Rat primary cardiomyocytes; USP, ubiquitin-specific protease.

Zhong et al. (2025), using a mouse model of atherosclerosis, found that the mitochondrial uncoupler (BAM15) reduced atherosclerotic plaque formation and decreased mtROS expression and oxidized mitochondrial DNA levels. Primary mouse aortic endothelial cells were isolated to validate the observation that BAM15 alleviates atherosclerosis by reducing endothelial pyroptosis via suppressing the expression of the NLRP3 inflammasome (Zhong et al., 2025). Tang F. et al. (2024) established an atherosclerosis model by using human aortic endothelial cells (HAEC) and ApoE^−/−^ C57BL/6 male mice, demonstrating that METTL3-mediated N6-methyladenosine (m6A) modification enhances long non-coding RNA (lncRNA) H19 stability, which, via the H19/NLRP3/Caspase-1 axis, promotes endothelial cell inflammation and pyroptosis, thereby exacerbating atherosclerosis (Tang F. et al., 2024).

Furthermore, endothelial cells and macrophages, as well as vascular smooth muscle cells, are also susceptible to pyroptosis. A bioinformatics analysis of target genes of ursolic acid revealed three critical genes—*IL1β*, *CASP1* and *CASP8*—by intersecting them with pyroptosis-related genes. The analysis revealed a close association between these genes, and *in vitro* experiments demonstrated that ursolic acid may serve as a possible inhibitor of pyroptosis in smooth muscle cells (MOVAS cells) during atherosclerosis (Huang et al., 2024).

### 3.2 Ischemic diseases

#### 3.2.1 Myocardial infarction

One of the most serious signs of coronary atherosclerotic heart disease is myocardial infarction, a cardiovascular disorder that is common around the world (Guo et al., 2024). Many molecular pathogenic pathways, including oxidative stress and inflammation, may contribute to the pathophysiology of myocardial infarction. Furthermore, the various stages of ischemic injury following the onset of myocardial infarction are associated with multiple types of programmed cell death, including apoptosis, necrotic apoptosis, ferroptosis, and pyroptosis (Xiang et al., 2024; Del Re et al., 2019; Piamsiri et al., 2023).

Retinol-binding protein 4 (RBP4) is an important pro-inflammatory adipokine (Yang et al., 2005). Emerging evidence has linked elevated circulating RBP4 levels to cardiovascular diseases, including atherosclerosis and coronary artery disease (Rychter et al., 2020). Zhang K. Z. et al. (2021) confirmed upregulation of RBP4 in the models of acute myocardial infarction (AMI). Its overexpression activates the NLRP3 inflammasome, promotes the caspase-1 precursor’s proteolysis, and subsequently induces GSDMD-dependent pyroptosis. RBP4 is therefore a novel regulator that promotes cardiomyocyte pyroptosis by interacting with the NLRP3 inflammasome in acute myocardial infarction.

Cui et al. (2025) revealed that AlkB homolog 5 (ALKBH5) influences m6A modification and messenger RNA (mRNA) stability of the NLRP3 inflammasome. The formation of this positive feedback loop thereby promotes cardiomyocyte pyroptosis following myocardial infarction. Therefore, inhibiting the ALKBH5-NLRP3 loop represents a new therapeutic approach and a potential target for the clinical treatment of cardiovascular diseases (Cui et al., 2025). Hypoxia-inducible factor (HIF)-1α exerts cardioprotective functions in myocardial infarction (Tekin et al., 2010). However, some reports conflict with these results, suggesting that HIF-1α also exerts a deleterious role in cardiac injury (Tang et al., 2008). Wang et al. (2022) found that Hypoxia/Reoxygenation (H/R) insult induced pyroptosis of cardiomyocytes, accompanied by increased expression of HIF-1α and TUG1. It has been established that HIF-1α interacts to TUG1’s promoter region to increase its expression, which causes the occurrence of AMI by encouraging mitochondrial damage and cardiomyocyte pyroptosis (Wang et al., 2022). Thus, Inhibition of the HIF-1α/TUG1/NLRP3 axis may represent a promising therapeutic target for mitigating cardiac injury.

#### 3.2.2 Myocardial ischemia-reperfusion injury

Among the leading causes of death worldwide, acute myocardial infarction ranks high (Heusch, 2020). Current research indicates that timely reperfusion therapies are critical for reducing mortality in acute myocardial infarction (Zhang et al., 2019; Keeley et al., 2003). However, for over 60 years, reperfusion has been paradoxically associated with myocardial cell death (via apoptosis, necrosis, and pyroptosis), energy metabolism disorders, and endothelial cell death resulting from coronary microvascular dysfunction—a phenomenon known as myocardial ischemia-reperfusion injury (Jeroudi et al., 1994). In recent years, the inflammatory cascade involving the NLRP3 inflammasome complex during myocardial ischemia-reperfusion injury has been extensively studied (Toldo et al., 2016; Silvis et al., 2021).

Ubiquitination/deubiquitination is an important form of post-translational protein modification that regulates the stability, intracellular localization, or activity of target proteins. In this process, E3 ubiquitin ligases are essential catalytic components that mediate substrate-specific identification and identify the kind of ubiquitination (Song et al., 2016; Liu Q. et al., 2019). *In vivo* research in mice overexpressing MARCH9, a membrane-tethered RING finger protein from the MARCH family of E3 ligases (Tan et al., 2019), revealed substantially decreased cardiac injury, smaller infarct areas, and lower inflammatory cell infiltration. Mechanistically, during ischemia-reperfusion, MARCH9 may cause K48-linked polyubiquitination of NLRP3 inflammasome, which inhibits NLRP3 inflammasome-mediated pyroptosis in cardiomyocytes (Lu H. et al., 2025). The deubiquitination process is regulated by deubiquitinating enzymes, mainly from the ubiquitin-specific protease (USP) family, which remove ubiquitin molecules from substrate proteins. The protective role of cardiac myocyte USP25 against myocardial ischemia-reperfusion injury has been reported recently. USP25 binds to the NLRP3 inflammasome and removes the K63-linked ubiquitin chain from the NLRP3 inflammasome; K63 is the activation tag for the NLRP3 inflammasome. Consequently, Inhibition of the NLRP3 inflammasome’s activation thereby affects the NLRP3-ASC interaction and ASC oligomerization, suppressing NLRP3 inflammasome activation and pyroptosis in cardiomyocytes (Ye et al., 2025).

Ozone promotes microcirculation, boosts tissue oxygen delivery, and has anti-inflammatory properties (Orakdogen et al., 2016). It has been found that pretreatment with ozone protects against myocardial ischemia-reperfusion damage. Rats are shielded against ischemia-reperfusion-induced cardiac damage by ozone’s anti-inflammatory effects, which are achieved by attenuating the NLRP3 inflammasome (Xu G. et al., 2025). Recently, researchers have also discovered that Up-frameshift protein 1 suppresses TXNIP/NLRP3-mediated pyroptosis in cardiomyocytes by degrading Kruppel-like factor 6, thereby alleviating myocardial ischemia-reperfusion injury (Yan et al., 2026).

#### 3.2.3 Ischemic stroke

About 85% of all strokes are ischemic strokes, which are a primary cause of death and disability worldwide (Iadecola and Anrather, 2011; Liu Z.-J. et al., 2019). Owing to limited treatment options and a narrow therapeutic window, many patients are unable to receive effective intervention and are at a high risk of severe neurological deficits and functional impairment (Mendelson and Prabhakaran, 2021). The pathogenesis of ischemic stroke mainly involves oxidative stress, neuronal death, and inflammation (Pan et al., 2022), with inflammation closely associated with the progression of ischemic injury. Recently, inflammasomes have been identified as key elements of ischemic stroke. Particularly, the NLRP3 inflammasome Pyrin domain is highly expressed in the brain and immune cells (Fann et al., 2013).

Recent evidence shows that serum levels of interferon-induced protein 35 are significantly elevated in patients with acute ischemic stroke. Furthermore, interferon-induced protein 35 levels were markedly increased in the serum and peri-infarct regions of mice with middle cerebral artery occlusion. Cell-based experiments have demonstrated that interferon-induced protein 35 exerts its effects through the TLR-4/NF-κB/NLRP3 axis to exacerbate BV2 microglial neuroinflammation under oxygen-glucose deprivation (OGD) conditions (Zhang M. et al., 2025). In both animal and cell culture experiments, Effusol has been shown to alleviate ischemic stroke by inhibiting the expression of TLR4 protein following induction of middle cerebral artery occlusion/reperfusion, thereby suppressing NLRP3 inflammasome activation (Xu L. et al., 2025). Adipose-derived stem cell-derived extracellular vesicles have been reported to inhibit the NLRP3/GSDMD pathway by suppressing Gbp3 expression, thereby improving neurological dysfunction and reducing inflammatory responses and pyroptosis (Wang J. et al., 2025). Differential gene expression analysis via GEO2R revealed significant upregulation of interferon-induced transmembrane protein 1 (IFITM1) in ischemic stroke cases, and demonstrated that IFITM1 knockdown could block the *in vitro* triggering of the NLRP3 inflammasome and reduce cerebral infarction and neurological damage in ischemic stroke (Li and Huang, 2025). Bakuchiol, found in the leaves and seeds of Psoralea corylifolia Linn, has been shown to reduce inflammation. Activation of the nuclear factor erythrocyte-related factor 2 (Nrf2) is crucial for inhibiting the NLRP3 inflammasome and downregulating pro-inflammatory cytokines to alleviate cerebral ischemia-reperfusion injury (Xu Y. et al., 2021). Subsequently, Xu Y. W. et al. (2025) used Nrf2 as a therapeutic target and performed middle cerebral artery occlusion/reperfusion (MCAO/R) in mice, and found that bakuchiol attenuated MCAO/R–induced cerebral infarction, suppressed the release of inflammatory factors, and reduced apoptosis and pyroptosis. Concurrent cellular experiments revealed that bacuchol alleviated cerebral infarction and hypoxia-reoxygenation injury by activating the adenosine monophosphate-activated protein kinase/Nrf2 (AMPK/Nrf2) pathway, which inhibits the TXNIP/NLRP3/Caspase-1 axis (Xu Y. W. et al., 2025).

*Toona sinensis fruit polyphenols* (TSFP) have demonstrated potential anti-inflammatory and neuroprotective properties. Through cerebral ischemia-reperfusion experiments in rats, Wang K. et al. (2025)’s team found that TSFP greatly enhanced neurological function and decreased the volume of brain infarcts. These effects occur via inhibiting p38 and ERK1/2 phosphorylation, promoting ERK5 phosphorylation, and suppressing the activation of the NLRP3/caspase-1/GSDMD axis, thereby alleviating pyroptosis and conferring neuroprotective benefits (Wang K. et al., 2025). Edaravone, a therapeutic agent for acute ischemic stroke (Zhang H. et al., 2025), has been validated in large-scale Phase II and III clinical trials to safely and effectively enhance patients with acute ischemic stroke’s 90-day functional outcomes (Xu J. et al., 2021; Fu et al., 2024). The underlying mechanism is that edaravone indirectly inhibits NLRP3 inflammasome activation and downstream signaling by suppressing STING, thereby alleviating neuroinflammation and limiting secondary damage following cerebral ischemia-reperfusion (Zhang Y. et al., 2025). A recent study further revealed that remimazolam alleviates cortical neuronal pyroptosis in rats by suppressing the NF-κB/NLRP3/caspase-1 axis, thus mitigating cerebral ischemia-reperfusion injury (Liu T. et al., 2025).

#### 3.2.4 Ischemic kidney disease

In recent years, with the accelerating population aging, the incidence of end-stage renal disease caused by ischemic kidney disease has been rising. Severe stenosis or obstruction of the renal artery leads to renal parenchymal damage and progressive renal dysfunction. The pathogenesis of ischemic renal disease involves various mechanisms of cell death and inflammatory responses (Ning et al., 2025), and accumulating evidence has revealed the role of pyroptosis, particularly NLRP3 inflammasome-mediated pyroptosis (Zhang B. et al., 2022; Zhang Y. et al., 2023; Wang et al., 2024).

Regulatory T cells (Tregs) are essentially involved in maintaining immune homeostasis and promoting self-tolerance (Sakaguchi, 2000; Sakaguchi et al., 2008). Patients with autoimmune and inflammatory diseases may benefit from increased Treg numbers and activity (Wright et al., 2009; Sharabi et al., 2018). CD8^+^ CD103+ iTregs act as protective components in the ischemia-reperfusion–acute kidney injury (AKI) process by regulating the NLRP3/Caspase-1 axis to control pyroptosis (Chen et al., 2024). Key regulators of cellular homeostasis are FOX transcription factors, especially FoxO3a. In kidney injury models, FoxO3a malfunction has been demonstrated to worsen cellular damage through a variety of mechanisms (Wu et al., 2018). Its upstream factor, methyltransferase G9a, catalyzes methylation of the lysine residue at position K262 of FoxO3a, thereby promoting recognition by the E3 ubiquitin ligase TRIM21. Subsequently, TRIM21 mediates K48-linked ubiquitination of FoxO3a at lysine 176, leading to its degradation and promoting the expression of NLRP3 inflammasome in ischemia/reperfusion injury–induced AKI kidneys (Zheng et al., 2025). m6A is the most prevalent epitranscriptomic modification in eukaryotic mRNA (Zhang L. et al., 2026). Emerging evidence suggests that dysregulation of m6A is broadly implicated in renal development and the advancement of kidney diseases, including renal cell carcinoma, AKI, and chronic kidney disease (Jung et al., 2024; Liu et al., 2024). METTL3 regulates a wide range of cell death pathways and is reported to promote TIFA expression in the 3′UTR through m6A modification; TIFA’s main function is to oligomerize and finish the NLRP3-Caspase-1 complex’s assembly. TIFA drives NLRP3 inflammasome activation and directly triggers the proteolysis of GSDMD (Ji et al., 2025). Recent studies have found that PRDM16 enhances the levels of USP10 by interacting with its promoter, thus inhibiting the levels of NLRP3 inflammasome and alleviating AKI caused by rhabdomyolysis (Han et al., 2025).

Dapagliflozin, a sodium-glucose cotransporter 2 inhibitor widely utilized in the therapy of diabetes and cardiovascular disease (Yoshihara et al., 2018; Petrie et al., 2020), has demonstrated efficacy in reducing renal inflammation and oxidative stress induced by limb ischemia-reperfusion. This protective effect is mediated by AMPK/SIRT1 pathway activation, which in turn inhibits pyroptosis by suppressing NLRP3 inflammasome activation (Zhu et al., 2025). In addition, pioglitazone, a thiazolidinedione drug typically used in the treatment of diabetes (Devchand et al., 2018; Richter et al., 2006), reduces inflammasome activation and improves cellular oxidative systems (Richter et al., 2006). Pioglitazone improves ischemia/reperfusion injury–induced AKI by inhibiting NLRP3 inflammasome activation (Ye Z. et al., 2024). Sodium aescinate is a natural blend of triterpene saponins containing ester bonds, widely utilized for treating post-traumatic, ischemia-reperfusion injury, and other conditions (Mei et al., 2023). Sodium aescinate has been shown to protect against renal ischemia-reperfusion injury, inhibit renal failure, and exert pyroptosis-inhibitory effects via suppression of the AKT-NLRP3 pathway (Xin L. et al., 2025).

#### 3.2.5 Ischemic retinal diseases

The retina is a highly oxygen-demanding tissue. Retinal ischemia/reperfusion may lead to retinal thinning, retinal ganglion cell death, and reduced electroretinogram activity (Li et al., 2019; Lee et al., 2021). Many ocular diseases, including diabetic retinopathy and glaucoma, are frequently brought on by retinal ischemia-reperfusion damage (Osborne et al., 2004). NLRP3 inhibitor MCC950 can reverse ischemic retinopathy by suppressing NLRP3 inflammasome activation (Sui et al., 2020). Using a rat model of ischemia-reperfusion injury, hydrogen sulfide alleviated retinal neuronal neuroinflammation by suppressing the NLRP3-Caspase-1-GSDMD pathway (Yang et al., 2022). Following retinal ischemia, Homer1 protects retinal ganglion cells by downregulating the TXNIP/NLRP3 pathway through the suppression of endoplasmic reticulum stress (Lv et al., 2023). Stmp1, a mitochondrial micropeptide encoded by a lncRNA, protected retinal ganglion cells from retinal ischemia-reperfusion injury in mice by attenuating the activation of the NLRP3 inflammasome pathway (Zheng et al., 2023).

### 3.3 Cardiomyopathy

#### 3.3.1 Dilated cardiomyopathy

Dilated cardiomyopathy (DCM), defined by left ventricular dilation and impaired contractile function (Weintraub et al., 2017), is a leading cause of heart failure. Its underlying mechanisms are highly complex and largely unclear, involving processes such as inflammation and apoptosis, which lead to cell death and ventricular remodeling, ultimately resulting in ventricular dilation and heart failure (Narula et al., 1996; Di Napoli et al., 2003).

In recent years, as pyroptosis has garnered increasing attention, a close link with DCM has been revealed. A study on DCM found that cardiac samples from DCM patients had ASC areas. Patients with DCM had considerably greater plasma levels of IL-18 and IL-1β than healthy controls, indicating that non-ischemic DCM patients’ cardiac tissue may activate the NLRP3 inflammasome (Zeng et al., 2020). Additionally, a study on DCM rats showed upregulation of histone deacetylases (HDACs), and HDAC6 knockdown enhanced cardiomyocyte autophagy, inhibited NLRP3 inflammasome activation, and alleviated cardiac injury in the myocardial tissues (Pang et al., 2023).

#### 3.3.2 Diabetic cardiomyopathy

As a common chronic disease, diabetes mellitus is estimated to affect approximately 783 million individuals worldwide by 2045 (Sun H. et al., 2022). Diabetic cardiomyopathy is considered an independent multifactorial condition leading to cardiac contractility impairment and ventricular hypertrophy (Lezoualc’h et al., 2023). According to recent research, pyroptosis contributes to the development and progression of diabetic cardiomyopathy (Liu et al., 2022; Yang et al., 2019).

UCP2 belongs to the mitochondrial uncoupling protein (UCP) family and is expressed in various tissues, including cardiac tissues (Murray et al., 2004). The level of NLRP3 inflammasome was reported to be elevated in the hearts of diabetic cardiomyopathy mice. The absence of UCP2 exacerbates ROS accumulation, leading to a further increase in TXNIP, which consequently triggers NLRP3 inflammasome activation, followed by GSDMD-mediated pyroptosis (Zhang L. et al., 2025). In addition, expression of the deubiquitinating enzyme USP13 was markedly downregulated in the cardiac tissue of diabetic cardiomyopathy mice. Mechanistic research verified that USP13 inhibits the NLRP3-ASC association by removing the K63-linked ubiquitin chain at K557 in the NLRP3 inflammasome, thereby suppressing ASC aggregation and activation of the NLRP3 inflammasome, eventually reducing pyroptosis in diabetic cardiomyocytes (Xu D. et al., 2025).

#### 3.3.3 Septic cardiomyopathy

Sepsis is a systemic inflammatory response syndrome triggered by infection (Cecconi et al., 2018). Sepsis-induced cardiomyopathy, which is characterized by reduced myocardial contractility, diastolic dysfunction, and myocardial cell death, is one of the most serious side effects of sepsis for which there is presently no specific treatment (Lv and Wang, 2016). Extensive necrotic cells and inflammatory cell infiltration have been identified in myocardial tissues from deceased sepsis patients (Hollenberg and Singer, 2021). This irreversible inflammatory cell death appears consistent with the characteristics of pyroptosis.

Necrotic-associated kinase 7 (NEK7), a serine/threonine kinase, can mediate the activation of the NLRP3 inflammasome (Sharif et al., 2019). NEK7 expression is upregulated in sepsis-induced cardiomyopathy mice, and its overexpression activates the NLRP3 inflammasome through NEK7-NLRP3 interaction, triggering pyroptosis (Wen et al., 2025). Traf2- and Nck-interacting kinase (TNIK) is a serine/threonine kinase (Shitashige et al., 2010). In a recent study, TNIK siRNA significantly downregulated TNIK and reduced NLRP3 inflammasome levels (Yang W. et al., 2025). As a potential therapeutic target for the management and prevention of sepsis-induced cardiomyopathy, TNIK shows promise. Interferon regulatory factor 1 is a potential upstream regulator of the NLRP3 inflammasome (Fujita et al., 1989; Kuriakose et al., 2018). Interferon regulatory factor 1, through NLRP3 inflammasome activation, induces LPS-induced myocardial injury, inflammation, and pyroptosis in mice (Pei et al., 2026).

### 3.4 Arrhythmias

Atrial fibrillation, a prevalent kind of sustained arrhythmia, is linked to a high risk of stroke, heart failure, and death (Chugh et al., 2014; Kirchhof et al., 2016). In 2018, Yao et al. (2018) first demonstrated the presence of functional NLRP3 inflammasomes in human atrial cardiomyocytes, a finding confirmed by Heijman et al. (2020). It has been shown that the specific NLRP3 inflammasome inhibitor MCC950 lessens atrial fibrosis and atrial fibrillation susceptibility (Zhang Y. et al., 2022).

A clinical trial revealed that in patients with non-valvular atrial fibrillation and heart failure with preserved ejection fraction (HFpEF), the expression of NLRP3 inflammasome, IL-1β, and B-type natriuretic peptide was positively correlated with the H2FPEF score and could serve as potential predictors of HFpEF in patients with non-valvular atrial fibrillation (Chen S. et al., 2025). Electrical remodeling in atrial fibrillation is associated with NLRP3 inflammasome activation (Zhang Y. et al., 2022). Hydrogen suppresses the activation of NLRP3 inflammasome by inhibiting NADPH oxidase (NOX) 4 expression and ROS production, thereby inhibiting electrical remodeling (Zhang B. et al., 2025) and improving atrial fibrillation and atrial fibrosis.

### 3.5 Heart failure

Heart failure represents the severe end-stage of various cardiac conditions (Savarese et al., 2017). The pathogenesis of heart failure is linked to cell death, inflammatory responses, myocardial fibrosis, and mitochondrial damage, with inflammatory responses playing a critical role in its progression (Qin et al., 2024). In recent years, pyroptosis, particularly NLRP3 inflammasome-induced pyroptosis, has proven crucial in the progression of heart failure.

In clinical research, researchers found reduced levels of Fat Mass and Obesity-associated protein (FTO) and increased levels of TLR4 in the fasting serum of individuals with heart failure. Subsequently, a myocardial cell injury model established using DOX validated that FTO mediates the m6A demethylation of TLR4 and regulates the binding activity of YTHDF1 to TLR4 mRNA, thereby downregulating TLR4. This, in turn, downregulates the NF-κB/NLRP3 pathway, inhibiting DOX-induced pyroptosis in cardiomyocytes (Tu et al., 2024). One of the primary causes of heart failure is oxidative stress (Ng et al., 2023), and ROS production is associated with the activation of macrophage inflammasomes, which may directly lead to cellular damage, oxidative stress, and pyroptosis (Zheng et al., 2022). NOX has traditionally been considered one of the most important proteins involved in ROS production in tissues and cells. NOX belongs to the flavin oxidase family, which includes numerous subtypes (NOX1–5) and dioxygenases 1 and 2. Dual oxidase 1 may promote caspase-1-dependent pyroptosis in AC16 cells by facilitating ROS production (Li et al., 2024).

Epicardial adipose tissue, a visceral fat reservoir located between the visceral pericardium and myocardium, can dynamically adapt to pathophysiological changes in the heart. It is a major source of pro-inflammatory adipokines, which can lead to myocardial fibrosis and microvascular dysfunction (Packer, 2018). Researchers using heart failure mouse and H9c2 cell models demonstrated that epicardial adipose tissue promotes inflammation by activating inflammasome-mediated pyroptosis signaling in adipocytes (Xia et al., 2022).

### 3.6 Pulmonary arterial hypertension

Pulmonary arterial hypertension (PAH) is a multifactorial chronic and progressive disease, characterized by elevated pulmonary artery pressure and pulmonary vascular remodeling, which ultimately leads to right heart failure and death (Vonk Noordegraaf et al., 2019; Price and Weatherald, 2023). Pyroptosis is inevitably closely linked to pulmonary arterial hypertension. The activation of macrophage-NLRP3 inflammasome has been reported to promote right ventricular failure in pulmonary arterial hypertension (Al-Qazazi et al., 2022).

In a mouse pulmonary hypertension model, the protein levels of NLRP3 inflammasome and cleaved caspase-1 were markedly elevated in the mouse lung tissue. Urolithin A inhibited NF-κB/NLRP3 signaling by activating AMPK, effectively preventing mice from developing pulmonary hypertension (He et al., 2024). Downregulation of the bone morphogenetic protein receptor 2 promotes the NLRP3/GSDME pathway, thereby inducing the inflammatory response associated with pulmonary arterial hypertension (Tian et al., 2025).

### 3.7 Myocarditis

Myocarditis is an inflammatory disease of the myocardium that may result from infection, be immune-mediated, or occur due to exposure to toxic substances (Ca et al., 2013; Lampejo et al., 2021). Mitsugumin 53, primarily expressed in cardiac muscle tissues, exerts cardioprotective effects through multiple mechanisms. Xue et al. (2024) reported that mitsugumin 53 negatively regulates NLRP3 inflammasome-mediated pyroptosis in coxsackievirus B3-induced acute viral myocarditis by inhibiting the NF-κB/NLRP3 axis in mouse HL-1 cardiomyocytes. Sun L. et al. (2022) found that the protective effect of IL-37 against cardiac coxsackievirus B3 infection may be involved in the inhibition of NF-κB/NLRP3 inflammasome/GSDMD. Cathepsin B, a lysosomal cysteine protease widely expressed in various cell types, exacerbates coxsackievirus B3-induced myocarditis by promoting the NLRP3/caspase-1 pathway (Wang et al., 2018).

In addition to viral myocarditis, the incidence of autoimmune myocarditis has been rising recently. Given the limited treatment options, its underlying mechanisms and effective therapeutic agents should urgently be identified. In an experimental autoimmune myocarditis mouse model developed by immunizing Balb/c mice with immunoglobulin fragments, Lupeol downregulated LACC1 expression via the peroxisome proliferator-activated receptor alpha, thereby inhibiting the NF-κB/NLRP3/GSDMD signaling pathways and exerting anti-inflammatory and anti-pyroptotic effects (Xiong et al., 2024).

### 3.8 Differential regulation of the NLRP3 inflammasome

Although various cardiovascular diseases are all associated with the NLRP3 inflammasome-mediated pyroptosis, their upstream regulatory mechanisms differ. The vessel walls in atherosclerotic arteries contain areas with complex geometries, which alter blood flow patterns and generate oscillatory shear stress (OSS) (Wang W. L. et al., 2025). This abnormal blood flow shear stress can trigger the IKKε-STAT1-NLRP3 signaling pathway, driving the transformation of atherosclerotic plaques from a stable phenotype to a high-risk necrotic phenotype (Lv et al., 2024). In addition, although both are ischemic conditions, in ischemic stroke, microglia are the first to be activated, releasing large amounts of inflammatory mediators, which subsequently recruit peripheral immune cells to the lesion site. The released inflammatory factors, such as IL-1β and IL-18, are released into the extracellular environment, triggering downstream MAPK and nuclear factor κB (NF-κB) pathways, leading to the transcription of IL-1β and IL-18 precursors as well as the NLRP3 inflammasome (Li et al., 2022), thereby exacerbating cerebral ischemia and reperfusion injury. Reactive oxygen species are produced in heart tissue when the oxygen supply to the myocardium is restored through reperfusion following a period of ischemia; these free radicals are directly associated with the activation of the NLRP3 inflammasome pathway (Shen et al., 2021). Endothelial dysfunction triggered by redox imbalance and the resulting activation of the NLRP3 inflammasome represent crucial early events that drive inflammatory responses in various cardiovascular diseases. Research has shown that direct NLRP3 inhibition has been demonstrated to exert a protective effect specifically on human coronary endothelial cells, rescuing them from oxidative and lipotoxic stress-induced injury (Penna and Pagliaro, 2025; Parenti et al., 2026). At the same time, during ischemia-reperfusion, in order to balance intracellular sodium concentrations, sodium-calcium exchangers transport excess intracellular sodium out of the cells and transport extracellular calcium into the cells, leading to calcium overload. Calcium overload results in mitochondrial instability and activation of the NLRP3 inflammasome (Shen et al., 2022). In sepsis-induced cardiomyopathy, the NLRP3 inflammasome is activated through multiple pathways; one of the most important upstream mechanisms is intracellular K+ depletion (Martínez-García et al., 2019); simultaneously, SIC leads to mitochondrial damage and the leakage of mtDNA, which can bind to the NLRP3 inflammasome and promote its activation (Chen et al., 2026). In diabetic cardiomyopathy, due to the hyperglycemic state, the thioredoxin-interacting protein (TXNIP) dissociates from thioredoxin and binds directly to the NLRP3 inflammasome; this is the most specific way that diabetic cardiomyopathy activates the NLRP3 inflammasome (Kakkar et al., 2025). In contrast, following CVB3 infection, inflammasome activation is driven by ROS and K^+^ efflux (Wang et al., 2014).

## 4 Potential applications of NLRP3 modulators

### 4.1 LncRNAs

These represent the non-protein-coding fraction of the human genome and have historically been regarded as junk DNA. However, over the past decade or so, the development of high-throughput technologies such as next-generation sequencing has facilitated deeper exploration of the non-coding genome (Kopp and Mendell, 2018). Research has shown that lncRNAs play a crucial role in cardiovascular disease (Dong et al., 2021) (Table 2).

**TABLE 2:** Potential applications of NLRP3 modulators.

Modulators		Disease	Models	Mechanism	Function (damage, protect)	References
LNCRNA	LncRNA Kcnq1ot1	Acute myocardial ischemia	H9c2	miR-27b-3p↓→NLRP3↑	Damage	Yang Y. et al. (2025)
	LncRNA GAS5	Myocardial fibrosis	Mouse/MCFs	mir-217↓→SIRT1↑→NLRP3↓	Protect	Zhang et al. (2024)
	LncRNA MHRT	Angiotensin II-induced cardiomyopathy	Mouse/AC16	Nrf2↑→ROS/NLRP3↓	Protect	Liu P. et al. (2023)
MIRNA	miR-223	Diabetic cardiomyopathy	H9c2	GRK2/NLRP3↑	Damage	Yang S. et al. (2025)
	MiR-155	Atherosclerosis	Mouse	MEK/ERK/NF-κB/NLRP3↑	Damage	Peng et al. (2022)
	miR-155-5p	Myocardial ischemia-reperfusion	H9c2	SIRT1↓→NLRP3↑	Damage	Lu Q. et al. (2025)
	miR-26a-5p	Atherosclerosis	Mouse	FoxO3a/ARC↓→NLRP3/GSDMD↑	Damage	Cai et al. (2025)
MSC-EXOs	MiR-199a-5p-containing EVs	Atherosclerosis	HAECS	SMARCA4↑→PODXL/NF-κB/NLRP3/caspase-1↓	Protect	Liang et al. (2023)
	miR-202-5p-containing EVs	Myocardial infarction	MCFs	TRAF3IP2/JNK/NLRP3↓	Protect	Chen J. et al. (2025)
	HIF1A-AS2-containing EVs	Atherosclerosis	Endothelial cells	miR-455-5p↓→ESRRG/NLRP3/caspase-1↑	Damage	Li P. et al. (2023)
Small-molecule inhibitors	DFV890	CHD	Human	IL-1β,IL-18↓	Protect	Novartis Pharmaceuticals (2023)
	colchicine	Myocardial infarction	Mouse/HL-1	ESR1/PI3K-Akt↑→NF-κB/NLRP3/GSDMD↓	Protect	Chen Y. et al. (2025)
Chinese herbs	Anatabine	Hypertension	Rat/PVN microglia	NF-κB/NLRP3/caspase-1↓	Protect	Su et al. (2025)
	Erianin	Autoimmune myocarditis	Mouse	NF-κB/NLRP3↓	Protect	Liu W. et al. (2025)
	*Allii macrostemonis* Bulbus	Atherosclerosis	Mouse/macrophage	NF-κB/NLRP3↓	Protect	Zhao et al. (2024)
	Sauchinone	Dilated cardiomyopathy	Mouse/H9c2	NLRP3↓	Protect	Xin L. et al. (2025)
	Paeoniflorin	Diabetic cardiomyopathy	Mouse/H9c2	AMPK/Nrf2↑→NLRP3↓	Protect	Zhang H. et al. (2026)
	BBR	Atrial fibrillation	Mouse	SIRT6-AMPK↑→NLRP3↓	Protect	Zhou et al. (2026)

Abbreviations: AMPK, adenosine monophosphate-activated protein kinase; BBR, berberine; HAECs, HAECs, human aortic endothelial cells; IL, interleukin; lncRNA, long non-coding RNA; miRNA, microRNA; MSC-EXOs, mesenchymal stem cell-derived exosomes; NF-κB, nuclear factor κB.

#### 4.1.1 LncRNA KCNQ1 overlapping transcript 1 (Kcnq1ot1)

Kcnq1ot1 is located on human chromosome 11p15.5 (Yang et al., 2018) and is a key mediator in cardiac disease. Inhibiting lncRNA Kcnq1ot1 can protect H9C2 cells from hypoxia-induced cellular damage by upregulating miR-27b-3p levels, thereby suppressing NLRP3 inflammasome (Yang Y. et al., 2025).

#### 4.1.2 LncRNA GAS5

Overexpression of GAS5 attenuates diabetic cardiomyopathy by inhibiting pyroptosis mediated by NLRP3 inflammasome activation (Xu et al., 2020). In particular, a recent study found that LncRNA GAS5 overexpression inhibits miR-217 expression, thereby promoting SIRT1 expression, which in turn suppresses myocardial fibrosis caused by NLRP3 inflammasome–mediated cardiomyocyte pyroptosis (Zhang et al., 2024).

#### 4.1.3 LncRNA MHRT

Overexpression of MHRT prevents Ang II-induced cardiomyocyte damage by activating Nrf2, reducing ROS accumulation, and inhibiting Ang II-induced oxidative damage in AC16 cardiomyocytes, as well as NLRP3 inflammasome activation (Liu P. et al., 2023).

### 4.2 microRNAs (miRNA)

A class of single-stranded, short non-coding RNAs (19–25 nucleotides) known as microRNAs (miRNAs) controls the expression of genes by attaching to the 3′-untranslated region (3′UTR) of target messenger RNAs (mRNAs) and causing either translational repression or mRNA destruction (Xu et al., 2022; Lu and Rothenberg, 2018).

#### 4.2.1 miR-223

Inhibiting miR-223–3p reduces the expression of G protein-coupled receptor kinase 2 and NLRP3 inflammasome, thus alleviating fibrosis and pyroptosis associated with diabetic myocardial injury (Yang S. et al., 2025).

#### 4.2.2 miR-155

miR-155 promotes atherosclerotic plaque formation by upregulating the MEK/ERK/NF-κB pathway in atherosclerotic plaques from ApoE-deficient mice, thus activating the NLRP3 inflammasome (Peng et al., 2022). Inhibiting miR-155-5p has recently been shown to suppress NLRP3 inflammasome activation by upregulating SIRT1 signaling, thereby alleviating H/R-induced pyroptosis in cardiomyocytes (Lu Q. et al., 2025).

#### 4.2.3 miR-26a-5p

It is abnormally expressed in various cardiovascular diseases (Kong et al., 2019). It can initiate autophagy, thereby activating the NLRP3 inflammasome, which in turn leads to increased cardiac hypertrophy (Tang L. Q. et al., 2024). Recently, researchers using Apoe −/− mice found elevated levels of miR-26a-5p in exosomes from all plaque regions within atherosclerotic tissue and demonstrated its effect in promoting NLRP3/GSDMD-mediated pyroptosis by inhibiting the FoxO3a/ARC axis (Cai et al., 2025).

### 4.3 Exosomes

Exosomes generated from mesenchymal stem cells (MSC-EXOs) show great promise for treating cardiovascular disorders by delivering various key components—including miRNAs, lncRNAs, and proteins—to inhibit cardiomyocyte death, promote angiogenesis, and alleviate inflammation (Liu Y. et al., 2023).

#### 4.3.1 MiR-199a-5p

Liang et al. (2023) demonstrated that macrophage-derived extracellular vesicles containing miR-199a-5p suppress the NF-κB/NLRP3/caspase-1 pathway by enhancing SMARCA4 signaling, thereby inhibiting PODXL expression in human aortic endothelial cells and reducing pyroptosis, which alleviates atherosclerosis.

#### 4.3.2 miR-202-5p

In a murine model of myocardial infarction, miR-202-5p transferred via induced pluripotent stem cell (iPSC)-MSC-EXOs inhibited NLRP3-mediated cardiomyocyte pyroptosis by downregulating the TRAF3-IP2/JNK pathway, thereby promoting cardiac function recovery (Chen J. et al., 2025).

#### 4.3.3 HIF1A-AS2

Extracellular vesicles released by endothelial cells deliver HIF1A-AS2, which can modulate miR-455-5p to upregulate estrogen receptor-related receptor gamma (ESRRG). Through the expression of ESRRG/NLRP3/caspase-1, this process triggers pyroptosis and vascular inflammation, thereby inducing atherosclerosis (Li P. et al., 2023).

### 4.4 Small-molecule inhibitors

Research on NLRP3 inhibitors has recently garnered significant attention, leading to the discovery of reversible and covalent NLRP3 inhibitors (Schwaid and Spencer, 2021; Bertinaria et al., 2019; Vong et al., 2021), such as MCC950 and VX-765. During acute myocardial ischemia, MCC950 and VX-765 attenuate myocardial injury by suppressing oxidative stress, inflammation, and pyroptosis (Ye X. et al., 2024). Mechanistically, MCC950 binds to the NACHT domain of the NLRP3 inflammasome, thereby inhibiting inflammasome activation. However, in a Phase II trial for rheumatoid arthritis, high doses (1,200 mg daily) led to severe hepatotoxicity in patients (ALT/AST levels exceeding three times the upper limit of normal), resulting in the trial’s termination. Its furan ring, which is metabolized by CYP450, may generate reactive intermediates; combined with the high dosage required, this exacerbates the risk of liver toxicity (Zhen et al., 2026). High-dose requirements, solubility limitations, and unpredictable off-target effects contribute to the complexity of safety evaluations for NLRP3 inhibitors. DFV 890 is a potent, selective, and highly bioavailable NLRP3 inflammasome antagonist that has demonstrated strong efficacy in pharmacokinetic/pharmacodynamic studies and in an acute mouse gout model. DFV890 underwent full Good Laboratory Practice (GLP) toxicology studies (in rats and macaques) and entered clinical trials in early 2019. Novartis has initiated a clinical study (NCT06031844) to evaluate the safety, efficacy, and tolerability of DFV890 in reducing inflammatory markers in participants with cardiovascular disease and elevated hsCRP (Novartis Pharmaceuticals, 2023). However, the outcomes of the efficacy endpoints of this study (such as changes in the inflammatory markers IL-6 and IL-18) have not yet been disclosed in any public database. The 1,3,4-oxadiazol-2-one derivative 5 (INF200) is a newly discovered NLRP3 inhibitor. *In vivo* studies have shown its effectiveness in ameliorating hyperlipoproteinemia-associated metabolic abnormalities, systemic inflammation, and myocardial dysfunction and in reducing ischemia-reperfusion injury–induced NLRP3 inflammasome activation and oxidative stress (Gastaldi et al., 2023).

Furthermore, existing drugs can be repurposed for the treatment of cardiac disease. Chen Y. et al. (2025) established an *in vivo* model of myocardial infarction in mice and found that colchicine could reduce inflammatory cell infiltration and fibrosis in the myocardium of mice with myocardial infarction, thereby improving cardiac function. They also revealed that colchicine alleviates pyroptosis in cardiomyocytes by acting on the estrogen receptor 1. In further mechanistic studies using HL-1 cells, colchicine suppressed cardiomyocyte pyroptosis following myocardial infarction by upregulating estrogen receptor 1–mediated PI3K-Akt signaling, thereby modulating the NF-κB/NLRP3/GSDMD axis (Chen Y. et al., 2025). Colchicine decreased the composite rate of cardiovascular death by 12% in a randomized controlled trial involving those who have experienced coronary heart disease in the past (d’Entremont et al., 2025). Therefore, colchicine can be used to treat unstable angina and prevent coronary heart disease, and as adjunctive therapy for myocardial infarction.

In the context of ischemic heart disease, pharmacological targeting of the NLRP3 inflammasome offers cardioprotective benefits, particularly by maintaining myocardial mechanical function and regulating redox pathways. Studies have shown that the recruitment and activation of the NLRP3 inflammasome are closely linked to redox stress: ROS drive NLRP3 inflammasome activation via the NF-κB pathway, and activated NLRP3 inflammasome, in turn, exacerbates oxidative stress (Pagliaro and Penna, 2023). This bidirectional positive feedback loop between ROS and the NLRP3 inflammasome forms the central hub of the “oxidative stress–inflammation” vicious cycle; targeting either end of this loop—whether by scavenging ROS or inhibiting the NLRP3 inflammasome—may effectively disrupt the cycle, offering a new intervention strategy for the treatment of cardiovascular diseases.

From this, we can see that the development of drugs targeting the NLRP3 inflammasome is crucial for the treatment of cardiovascular diseases; however, the process of translating findings from animal models to human trials is fraught with challenges. The key to achieving therapeutic efficacy *in vivo* lies in ensuring that inhibitors reach sustained and effective exposure levels in target tissues (such as the brain and blood vessel walls) and reliably inhibit NLRP3 inflammasome function. However, the relationship between the cerebrospinal fluid penetration and tissue residence time of most compounds under investigation and the degree of inhibition of downstream pharmacodynamic biomarkers (such as IL-1β and IL-18) has not yet been clearly established in humans. The lack of robust PK/PD models makes clinical dose exploration akin to a blind man feeling an elephant. Second, increasing clinical doses raises the risk of adverse reactions. Striking a precise balance between efficacy and safety, and developing molecules with a broader therapeutic window or enhanced tissue specificity, will be a key focus in the future. At the same time, our research in animal models has largely been limited to administration following acute injury, rather than the “treatment” of chronic, progressive human diseases—which is the most critical underlying factor contributing to clinical trial failures. In summary, the future success of clinical trials for NLRP3 inhibitors depends not only on safer and more effective new molecules but also, more urgently, on resolving these fundamental translational issues—particularly the establishment of a robust PK/PD/biomarker triangulation validation system.

### 4.5 Chinese herbs

#### 4.5.1 Anatabine

It is found in Solanaceae plants, including tobacco, tomatoes, chili peppers, and eggplants, and possesses potent anti-inflammatory properties (Dewey and Xie, 2013). By blocking the NF-κB/NLRP3/caspase-1 pathway in PVN microglia, anatabine has been found to successfully lower blood pressure, decrease sympathetic drive, and prevent heart structural remodeling (Su et al., 2025).

#### 4.5.2 Erianin

It is a phytoestrogen with therapeutic potential and one of the primary active components of Dendrobium (Li G. et al., 2023). Erianin possesses a broad and potent pharmacological profile, including antitumor and anti-inflammatory (Zhang X. et al., 2021; Yang et al., 2023). Furthermore, it alleviates autoimmune myocarditis by downregulating the NF-κB/NLRP3 signaling pathway (Liu W. et al., 2025).

#### 4.5.3 Allium macrostemonis bulbus

It is a Chinese herb with medicinal and food homology, mainly comprising steroidal saponins (Yao et al., 2016). In high-fat diet-fed ApoE^−/−^ mice, oral administration of Allium macrostemonis bulbus significantly reduced inflammation and formation of atherosclerotic plaques. *In vitro* experiments indicated that this beneficial action is partly mediated via regulation of the NF-κB/NLRP3 pathway, exhibiting potential anti-atherosclerotic effects (Zhao et al., 2024).

#### 4.5.4 Sauchinone

It is a non-antiparallel isomer of xylone isolated from Ziziphus jujuba, known for its anti-inflammatory effects (Bae et al., 2010). Sauchinone alleviated Dox-induced cardiac damage and remodeling in mice. Additionally, *in vitro* experiments revealed that sauchinone prevented Dox from activating the NLRP3 inflammasome in H9c2 cells and reduced Dox-induced cardiac inflammatory infiltration (Xin W. et al., 2025).

#### 4.5.5 Paeoniflorin

This monoterpenoid glycoside is the major bioactive component of Paeoniae radix Rubra (Zhang X.-X. et al., 2022). A recent study showed that paeoniflorin improved myocardial fibrosis and hypertrophy in a diabetic model; *in vitro* experiments demonstrated its inhibitory effect on NLRP3-mediated inflammatory responses and pyroptosis by upregulating AMPK/Nrf2 signaling (Zhang H. et al., 2026).

#### 4.5.6 Berberine (BBR)

This plant-derived isoquinoline alkaloid could inhibit inflammatory responses (Ahmedy et al., 2023; Wang et al., 2023). Berberine prevents atrial fibrillation by promoting the SIRT6-AMPK axis to inhibit NLRP3 inflammasome signaling (Zhou et al., 2026).

## 5 Clinical significance of peripheral blood

The diagnosis and treatment of cardiovascular diseases depend heavily on the NLRP3 inflammasome. Compared with healthy controls, patients with diabetes exhibit elevated levels of NLRP3 inflammasome in peripheral blood mononuclear cells and IL-1β in plasma, which are positively related to the severity of atherosclerosis (Wan et al., 2019). Thus, the NLRP3 inflammasome may act as a promising biomarker for atherosclerosis in diabetes. The triglyceride-to-glucose ratio (TyG ratio) is an additional biomarker closely associated with cardiovascular health (Zhao et al., 2022). Data from 100 patients with myocardial infarction were analyzed in a clinical trial, which revealed that the case group exhibited significantly elevated levels of triglycerides, fasting plasma glucose (FPG), and TyG index compared with the control group. Additionally, the observation group had significantly higher levels of the inflammatory marker NLRP3 inflammasome. Patients with elevated NLRP3 inflammasome and TyG ratio had poorer prognoses, manifested by deteriorating heart health and a higher chance of cardiovascular events (Yao et al., 2024). These findings highlight the significance of the NLRP3 inflammasome index as a potential biomarker of poor prognosis in cardiac ischemia-reperfusion injury.

## 6 Issues and outlook

Numerous cardiovascular system cells exhibit the NLRP3 inflammasome, but there are fundamental differences in its function and pathogenic significance; macrophages are the primary source of IL-1β in atherosclerosis. Inhibiting the NLRP3 inflammasome in macrophages can effectively reduce plaque inflammation. Activation of the NLRP3 inflammasome impairs the function of vascular endothelial nitric oxide synthase, promoting endothelial-to-mesenchymal transition and a procoagulant phenotype. Selective inhibition of the NLRP3 inflammasome in vascular endothelial cells may offer additional benefits in terms of improved vasodilation and antithrombotic effects; however, it remains unclear whether existing inhibitors can effectively penetrate endothelial cells. Future research should utilize conditional knockout animal models and cell-type-specific delivery systems, thereby guiding more precise targeting strategies.

Currently, most NLRP3 inhibitors are administered systemically via oral or injectable routes, which raises significant safety concerns. Long-term systemic inhibition of the NLRP3 inflammasome can impair the body’s response to danger signals. Systemic immunosuppression may disrupt the innate immune response after immunization or raise the risk of opportunistic infections in older people with cardiovascular disease. The NLRP3 inflammasome is an important regulator of the aging-associated secretory phenotype. Since cardiovascular patients are often elderly, long-term systemic inhibition of the NLRP3 inflammasome may slow inflammatory aging but could also suppress the immune surveillance system’s ability to clear senescent cells; its long-term net benefit remains unknown. To mitigate these risks, strategies that confine the therapeutic window to specific regions of the blood vessels or heart are highly attractive. For example, nanoparticles that respond to the inflammatory microenvironment of plaques (acidic pH, reactive oxygen species) or drug conjugates coupled to myocardial cell-specific ligands can be used to achieve localized accumulation and release of NLRP3 inhibitors at the lesion site. In addition, inhalation administration or intracoronary local perfusion may also reduce systemic exposure. This requires the identification of measurable indicators of drug efficacy within the affected tissue and of systemic immune function.

In Section 5, we discussed the value of peripheral blood NLRP3 inflammasome or its downstream products as biomarkers; however, blood markers can, by their very nature, only reflect the systemic inflammatory burden and cannot precisely pinpoint the location of the lesion. Plasma IL-1β and IL-18 have short half-lives and are susceptible to circadian rhythms and transient infections, which means that blood biomarkers may yield false-negative results or show a lag when evaluating drug efficacy. Ideally, the activation status of the NLRP3 inflammasome in the diseased tissue should be detected directly. However, the invasive nature of local vascular sampling limits its feasibility and reproducibility. Therefore, future efforts could focus on developing positron emission tomography (PET) tracers that target the NLRP3 inflammasome or active caspase-1 to enable *in vivo*, noninvasive, dynamic visualization of plaques or myocardial inflammation, thereby directly confirming target binding and the extent of inflammation suppression.

Furthermore, the progression of ischemic heart disease and the accompanying activation of the NLRP3 inflammasome do not occur uniformly in all individuals but are significantly modulated by key clinical variables such as age and sex. Researchers evaluated the effects of aging and sex differences on the NLRP3 inflammasome pathway and found that older individuals and male animals exhibited more pronounced NLRP3 inflammasome activation and poorer outcomes following ischemic injury, which is associated with age-related mitochondrial dysfunction and the differential regulation of the NF-κB pathway by androgens (Alloatti et al., 2022). This implies that the efficacy of NLRP3-targeted therapies in clinical translation may vary depending on patient age and sex; future clinical trials should be designed to fully account for these biological variables.

As a central link between inflammatory responses and cardiovascular disease, research on NLRP3 inflammasome-mediated pyroptosis has achieved breakthrough progress, revealing entirely new perspectives for understanding disease pathogenesis and developing novel therapeutic strategies. However, this field is still unexplored, with numerous critical scientific questions awaiting in-depth research. These include clarifying the cell- and tissue-specific mechanisms underlying its role in disease, unraveling complex molecular regulatory networks, and overcoming key bottlenecks in clinical translation. Future research is likely to focus on in-depth mechanistic analysis and multidimensional exploration to construct a more comprehensive disease network. Meanwhile, the development of highly effective and specific NLRP3 inflammasome inhibitors represents a core future direction; however, long-term safety, tissue specificity, and off-target effects still require rigorous evaluation. Future drug design should emphasize targeted delivery systems (such as nanocarriers targeting diseased blood vessels or cardiomyocytes) to improve treatment outcomes while minimizing adverse effects linked to systemic immunosuppression.

In summary, targeting NLRP3-mediated pyroptosis represents a promising new frontier in the treatment of cardiovascular disease. Through interdisciplinary collaboration and in-depth research spanning from molecular mechanisms to clinical translation, we can anticipate the development of novel “anti-inflammatory” therapies centered on pyroptosis inhibition, thereby providing a more efficacious therapeutic strategy against cardiovascular disease.
